# 5-Fluorouracil and folinic acid: interesting biochemistry or effective treatment?

**DOI:** 10.1038/bjc.1989.370

**Published:** 1989-12

**Authors:** D. J. Kerr


Br. J. Cancer (1989), 60, 807-808 © The Macmillan Press Ltd., 1989

GUEST EDITORIAL

### 5-Fluorouracil and folinic acid: interesting biochemistry or effective treatment?

D.J. Kerr

*CRC Dept of Medical Oncology, 1 Horselethill Road, University of Glasgow, Glasgow G12 9LX, UK.*

While waiting for the introduction of novel, active cytotoxic agents to the clinic, it behoves us to utilise currently available anti-neoplastic drugs as best we can. This implies that we continue to extend knowledge of the drug's clinical and cellular pharmacology and optimise drug delivery in terms of dose, route of administration, schedule, combination with other agents, and circadian ordered timing of administration. The addition of folinic acid (leucovorin, etc.) to 5-fluorouracil is one such area where knowledge of the drugs mechanism has led to proposals for its more effective use in the clinic.

In essence, 5-Fu is metabolised to 5-dUMP which binds to and inhibits the enzyme thymidylate synthetase (Figure 1). The ability of 5-dUMP to inhibit the enzyme is enhanced by the presence of 5,10-$CH_2$-$FH_4$ which can by synthesised from FA, and stabilises the formation of a covalent, ternary complex of F-dUMP with thymidylate synthetase (Figure 1).

There are extensive preclinical data, both *in vitro* and *in vivo* (Houghton *et al.*, 1982) suggesting that the combination of 5-Fu/FA is significantly more efficacious than 5-Fu alone. In order to provide guidelines for clinical usage, *in vitro* studies have been used to define minimum effective concentrations and durations of exposure to FA required to maximise the cytotoxicity of 5-Fu. For example, the lowest concentration of FA that provided maximum potentiation was 10 μM for mouse sarcoma 180 cells treated with 5-Fu at a concentration of 30 μM for 3 h. This implies that attempts should be made to achieve plasma FA concentrations of at least 10 μM and maintain these for at least 3 h (Evans *et al.*, 1981). This type of approach may seem naive, in view of the complex intracellular metabolism which both drugs must undergo to be 'activated' and as plasma drug concentrations at best given an indirect estimate of drug concentration at its molecular target. Nevertheless, this allows us to use the clinical pharmacokinetic parameters for FA to calculate dose regimens which will achieve 'ball-park' plasma exposures compared to those considered active *in vitro*, and must serve as a more scientific method of deriving dose schedules than empiricism.

There has been a large number of phase I/II studies of 5-Fu in combination with FA using a range of dose schedules (for both drugs). Clinical studies have focused mainly on those tumour types in which 5-Fu has a defined role such as gastro-intestinal, breast and squamous cell carcinoma of head and neck.

Machover *et al.* (1986) performed a phase II trial of 5-Fu/FA in patients with advanced gastric carcinoma. In previously treated patients (26 out of 27 available patients), one CR and 12 PRs were seen (50% objective response rate), with a median time to disease progression of almost 6 months. Other phase II studies with different dose schedules show activity, but at lower levels. There are no mature phase III trials in gastric carcinoma. Phase II studies in advanced breast cancer, some including patients previously treated with 5-Fu containing regimens, generally indicate a range of useful activity for 5-Fu/FA. Doroshow *et al.* (1989) have shown that 5-Fu (bolus 370 mg $m^{-2}$ $day^{-1}$ on days 1-5) and high dose folinic acid infusion (500 mg $m^{-2}$ $day^{-1}$ on days 1-6) is a useful salvage therapy in patients with refractory metastatic breast cancer. Sixty patients, who had been previously treated with 5-Fu containing regimens, were treated with 5-Fu/FA and 1 CR (duration 8.7 months) and 9 PRs (median duration 3.2 months) were seen (objective response, 17%, 95% confidence intervals for response, 8-27%).

**Figure 1** Intracellular metabolism of 5-fluorouracil (5-FU) and folinic acid (FA). FdUrd, fluorodeoxyuridine; FdUMP, fluorodeoxyuridine monophosphate; dUMP, deoxyuridine monophosphate; dTMP, thymidylate; 5,10-$CH_2$-$FH_4$, 5,10-methenyl-tetrahydrofolate; $FH_2$, dihydrofolate; 5-$CH_3$-$FH_4$, 5-methyltetrahydrofolate; $FH_4$, tetrahydrofolate.

There is limited evidence suggesting that 5-Fu/FA is active in locally advanced squamous carcinoma of the head and neck, but it has already been incorporated into complex split chemotherapy/radiotherapy regimens for this disease (Wendt *et al.*, 1989).

The most mature data, in terms of phase III trials, have been accrued for threatment of patients with advanced colorectal carcinoma. An update of current results from seven randomised trials comparing 5-Fu/FA against single agent 5-Fu was presented recently by O'Connell (1989) at the NCI-EORTC symposium on new cancer drugs (Amsterdam, March 1989). Although the dose and treatment schedules varied, 1,058 patients had been randomised and six of the seven trials have shown a significantly higher response rate in the 5-Fu/FA arms. Interestingly two of the trials (O'Connell,

Received 14 August 1989.

1989; Erlichman *et al.*, 1986) with similar dose schedules (bolus 5-Fu, 370–425 mg $m^{-2}$ $day^{-1}$ on days 1–5; bolus FA, 20 or 200 mg $m^{-2}$ $days^{-1}$ on days 1–5) demonstrated significant prolongation of overall survival in the 5-Fu/FA arm (median survival 1 year *vs* 7.5 months). Future randomised studies in advanced colo-rectal carcinoma will compare the Roswell Park Memorial schedules (5-Fu bolus of 600 mg $m^{-2}$ at the mid point of a 2 h infusion of FA, 500 mg $m^{-2}$ repeated weekly × 6) against the Mayo clinic/NCCTG schedule (with low dose FA, 20 mg $m^{-2}$). The results of the randomised phase III studies provide a strong scientific rationale for the use of 5-Fu/FA combinations as surgical adjuvant therapy for patients with colorectal carcinoma.

Dose limiting toxicity of the 5-Fu/FA combination depends on the schedule. It tends to be severe diarrhoea for weekly administration and mylosuppression, and stomatitis for the 5 × daily loading course. Certainly in the Mayo Clinic/NCCTG experience, stomatitis was worse in the combination arm, diarrhoea was mild and evenly distributed and myelosuppression was more marked with 5-Fu alone. A quality of life assessment indicated that 5-Fu plus low dose FA was the preferred treatment.

Is this treatment cost effective? According to our current hospital pharmacy prices, a 6-week course of 5-Fu/FA according to the Roswell Park Memorial Schedule would cost approximately £540.00 compared to £15.00 for 5-Fu. However, response rates and survival were similar in the Mayo Clinic/NCCGT study comparing two different FA schedules (200 mg $m^{-2}$ $day^{-1}$ on days 1–5 versus 20 mg $m^{-2}$ $day^{-1}$ on days 1–5) and therefore one would conclude from this trial that 5-Fu/low dose FA was the preferred arm in terms of cost effectiveness.

Is it possible to refine combination treatment with 5-Fu/FA further? The notion of having a specific, measurable biochemical target (the ternary complex with thymidylate synthetase) for 5-Fu/FA implies an ability to titrate drug dose schedules almost to an individual level, if there are correlates between response and tissue biochemical parameters. Investigators at the NCI and Mayo Clinic are attempting, prospectively, to estimate the kinetics of formation of the thymidylate synthetase–F-dUMP complex using a radioimmuno assay in sequential breast cancer biopsies following treatment with 5-Fu/FA, and relate this to response. This is a fascinating study and obviously, potentially gives much more information than measuring plasma drug concentrations.

In terms of 5-Fu delivery, there is increasing evidence to suggest that prolonged, continuous infusions of 5-Fu yield higher response rates in metastatic colorectal cancer than conventional bolus (weekly or loading) administration. Lokich *et al.* (1989) have recently reported that continuous infusion of 5-Fu, 300 mg $m^{-2}$ $day^{-1}$ for 10 weeks had a significantly higher response rate than bolus 5-Fu, 500 mg $m^{-2}$ $day^{-1}$ on days 1–5 (30% *vs* 7%). It may be that prolonged exposure to 5-Fu leads to stabilisation or a greater degree of inhibition of thymidylate synthetase than generating high intracellular drug concentration for brief periods following bolus administration (5-Fu has a plasma half-life of approximately 10 min). Obviously, there may be a potential to combine prolonged infusions of 5-Fu with FA.

This is an interesting field, and we all like to see 'old drugs learn new tricks', but it is important to remember that the survival benefits of combined treatment, when conferred, are, thus far, relatively minor and this treatment combination should remain the subject of further research rather then routine clinical application.

### References

DOROSHOW, J.H., LEONG, L., MARGOLIN, K. *et al.*, (1989). Refractory metastatic breast cancer: salvage therapy with fluorouracil and high-dose continuous infusion leucovorin calcium. *J. Clin. Oncol.*, **7**, 434.

ERLICHMAN, C., FINE, S., WONG, A. *et al.* (1986). A comparison of 5-fluorouracil (5FU) and folinic acid (FA) versus 5FU in metastatic colorectal carcinoma (MCC). *Proc. ASCO*, **5**, 82.

EVENS, R.M., LASKIN, J.D. & HAKALA, M.T. (1981). Effects of excess folates and deoxyinosine on the activity and site of action of 5-fluorouracil. *Cancer Res.*, **41**, 3288.

HOUGHTON, J.A., SCHMIDT, C. & HOUGHTON, P.F. (1982). The effect of derivatives of folic acid on the fluorodexoyuridylate-thymidylate synthetase covalent complex in human colon xenografts. *Eur. J. Cancer Clin. Oncol.*, **18**, 347.

LOKICH, J.J., AHLGREN, J.D., GULLO, J.J., PHILIPS, J.A. & FRYER, J.F. (1989). A prospective randomised comparison of continuous infusion fluorouracil with a conventional bolus schedule in metastatic colorectal carcinoma: a mid-Atlantic oncology study program. *J. Clin. Oncol.*, **7**, 425.

MACHOVER, D., GOLDSCHMIDT, E., CHOLLET, P. *et al.*, (1986). Treatment of advanced colorectal and gastric adenocarcinomas with 5-fluorouracil and high-dose folinic acid. *J. Clin. Oncol.*, **4**, 685.

O'CONNELL, M.J. (1989). Results of phase III trials of 5-FU/leucovorin in treatment of advanced colo-rectal carcinoma. Proc. Sixth NCI/EORTC New Drugs Symposium, Amsterdam.

WENDT, T.G., HARTENSTEIN, R.C., WUSTROW, T.P.U. & LISSNER, J. (1989). Cisplatin, fluorouracil with leucovorin calcium enhancement, and synchromous accelerated radiotherapy in the management of local advanced head and neck cancer: a phase II study. *J. Clin. Oncol.*, **7**, 471.


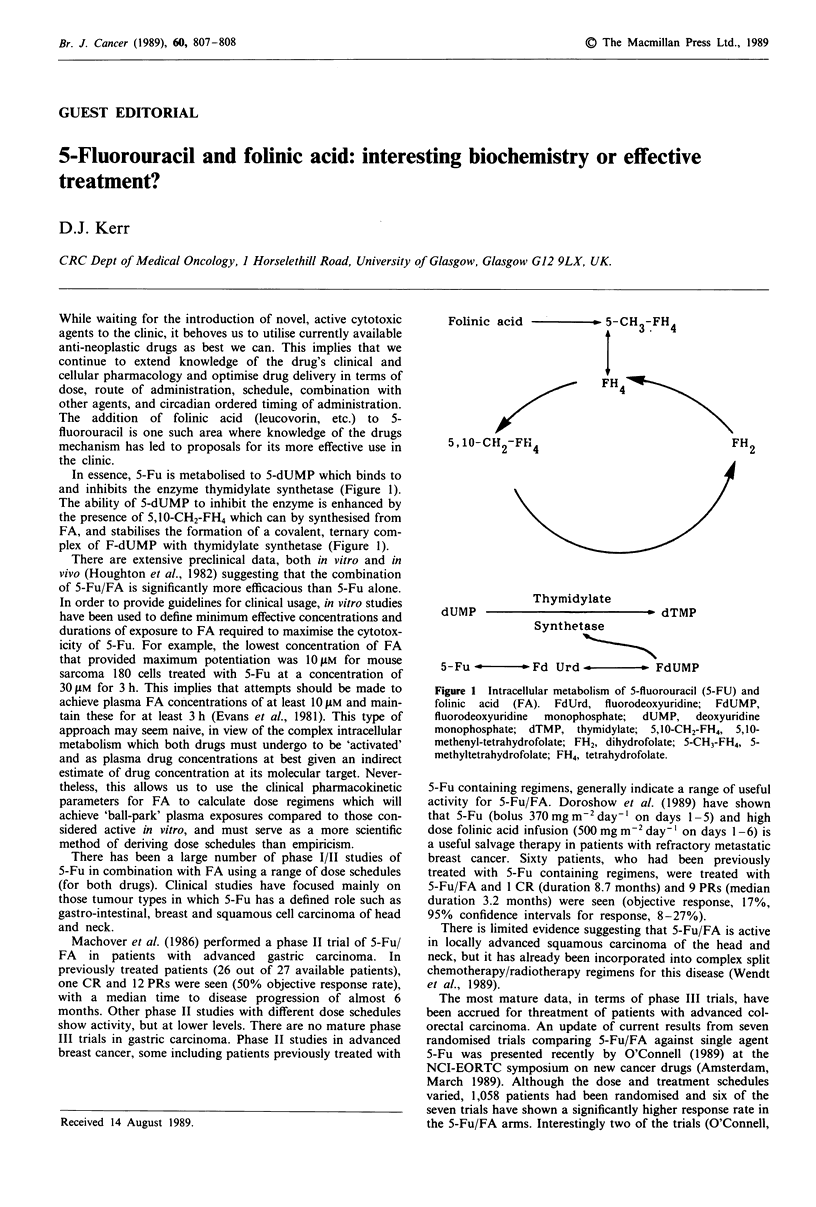

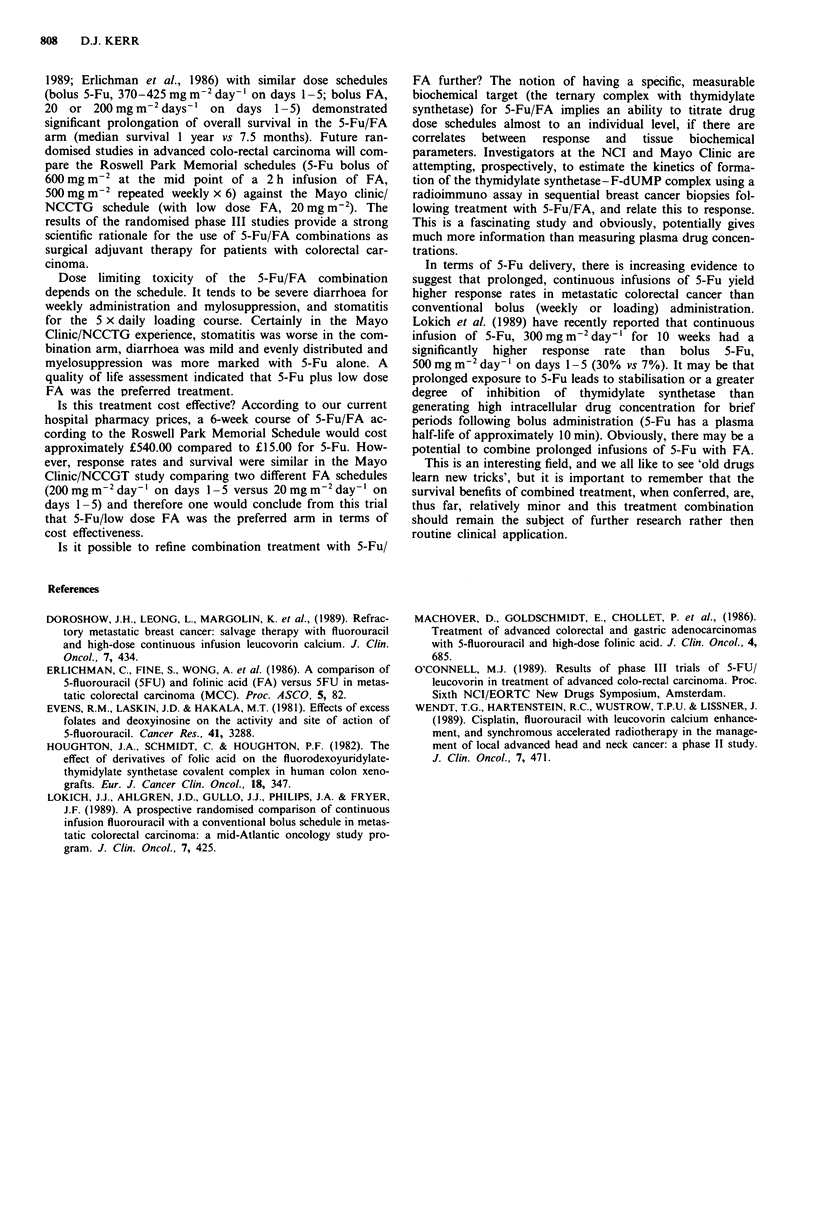


## References

[OCR_00220] Evans R. M., Laskin J. D., Hakala M. T. (1981). Effect of excess folates and deoxyinosine on the activity and site of action of 5-fluorouracil.. Cancer Res.

[OCR_00225] Houghton J. A., Schmidt C., Houghton P. J. (1982). The effect of derivatives of folic acid on the fluorodeoxyuridylate-thymidylate synthetase covalent complex in human colon xenografts.. Eur J Cancer Clin Oncol.

[OCR_00231] Lokich J. J., Ahlgren J. D., Gullo J. J., Philips J. A., Fryer J. G. (1989). A prospective randomized comparison of continuous infusion fluorouracil with a conventional bolus schedule in metastatic colorectal carcinoma: a Mid-Atlantic Oncology Program Study.. J Clin Oncol.

[OCR_00238] Machover D., Goldschmidt E., Chollet P., Metzger G., Zittoun J., Marquet J., Vandenbulcke J. M., Misset J. L., Schwarzenberg L., Fourtillan J. B. (1986). Treatment of advanced colorectal and gastric adenocarcinomas with 5-fluorouracil and high-dose folinic acid.. J Clin Oncol.

[OCR_00249] Wendt T. G., Hartenstein R. C., Wustrow T. P., Lissner J. (1989). Cisplatin, fluorouracil with leucovorin calcium enhancement, and synchronous accelerated radiotherapy in the management of locally advanced head and neck cancer: a phase II study.. J Clin Oncol.

